# Postcoital Urethral and Penile Trauma in a 28-Year-Old Male: A Case Report and Surgical Management

**DOI:** 10.7759/cureus.87572

**Published:** 2025-07-08

**Authors:** Stanislaw Szymkiewicz

**Affiliations:** 1 Department of Urology, Janusz Korczak Provincial Specialist Hospital, Slupsk, POL

**Keywords:** case report, corpus cavernosum rupture, penile fracture, sexual trauma, urethral injury, urethrorrhagia

## Abstract

Urethral bleeding following sexual activity is uncommon and typically benign. However, in rare cases, it may indicate significant trauma to the penile structures requiring surgical intervention. A 28-year-old male was admitted to the Urology Department from the Emergency Department due to profuse urethral bleeding after sexual intercourse. Physical examination revealed blood at the external urethral meatus, but no penile deviation, hematoma, or swelling. This atypical presentation without external signs of trauma delayed definitive diagnosis. Due to persistent bleeding and suspected cavernosal and urethral injury, the patient was qualified for surgical exploration. Intraoperatively, through a penoscrotal approach, complete denudation of the penis was performed. A tourniquet was placed at the penile base. Saline injection into the corpora cavernosa revealed a tear on the ventral aspect of both the corpus spongiosum and cavernosa. Buck’s fascia was dissected, and the urethral injury was repaired with 5-0 Monocryl absorbable sutures. The cavernosal rupture was closed with 2-0 Serapren. A saline leak test confirmed a watertight repair. Layered closure was performed, a Foley catheter was inserted, and a sterile dressing was applied. Although rare, combined rupture of the urethra and corpora cavernosa following sexual intercourse can occur and requires prompt diagnosis and surgical repair. Penile exploration with layered reconstruction ensures good functional and cosmetic outcomes.

## Introduction

Penile trauma associated with sexual activity is a urological emergency, most commonly presenting as a penile fracture. While classic penile fracture involves rupture of the tunica albuginea of the corpora cavernosa, concurrent urethral injury is less frequent, seen in approximately 10%-20% of cases [1,2]. Prompt surgical repair is crucial to prevent long-term complications such as erectile dysfunction, urethral stricture, or penile curvature [3,4]. This case report presents an unusual presentation of postcoital bleeding due to combined rupture of the corpus spongiosum, corpus cavernosum, and urethra in a young male, managed surgically with a favorable outcome.

## Case presentation

A 28-year-old previously healthy male presented to the Emergency Department with acute urethral bleeding following consensual sexual intercourse. He reported hearing a sudden "cracking" sound followed by immediate penile detumescence and active bleeding from the urethral meatus. On examination, the penis appeared externally normal, without swelling, hematoma, or deviation. The only clinical finding was active bleeding from the urethral meatus. Blood was noted at the urethral orifice. No scrotal tenderness or perineal ecchymosis was present. Laboratory tests, including urinalysis, CBC, and CRP, were unremarkable. Due to clinical suspicion of underlying corporal and urethral injury, the patient was taken to the operating room.

Surgical procedure

**Figure 1:**
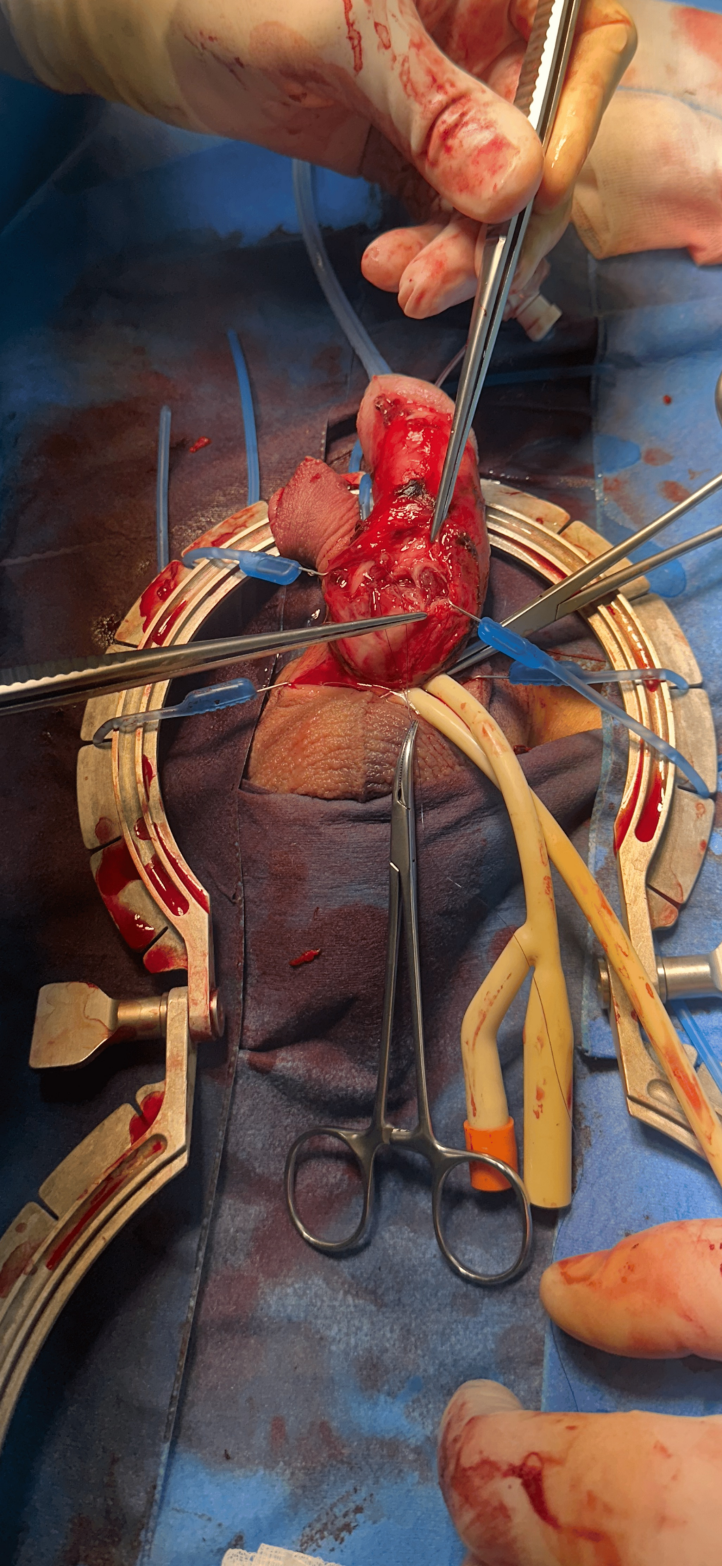
Intraoperative image showing rupture of the corpus spongiosum, corpus cavernosum, and urethra

Sterile field preparation was performed, followed by penile degloving via a penoscrotal approach. A tourniquet was applied at the penile base. Injection of 0.9% NaCl into the corpora cavernosa revealed leakage from a tear on the ventral side of the penis involving both the corpus spongiosum and urethra. The deep (Buck's) fascia was dissected. Urethral repair was performed using 5-0 Monocryl absorbable sutures. Tunical repair of the corpus cavernosum was completed with 2-0 Serapren sutures. A repeat saline test confirmed a watertight closure. Layered penile closure was carried out. A Foley catheter was inserted and a sterile dressing applied (Figure 1).

**Figure 2:**
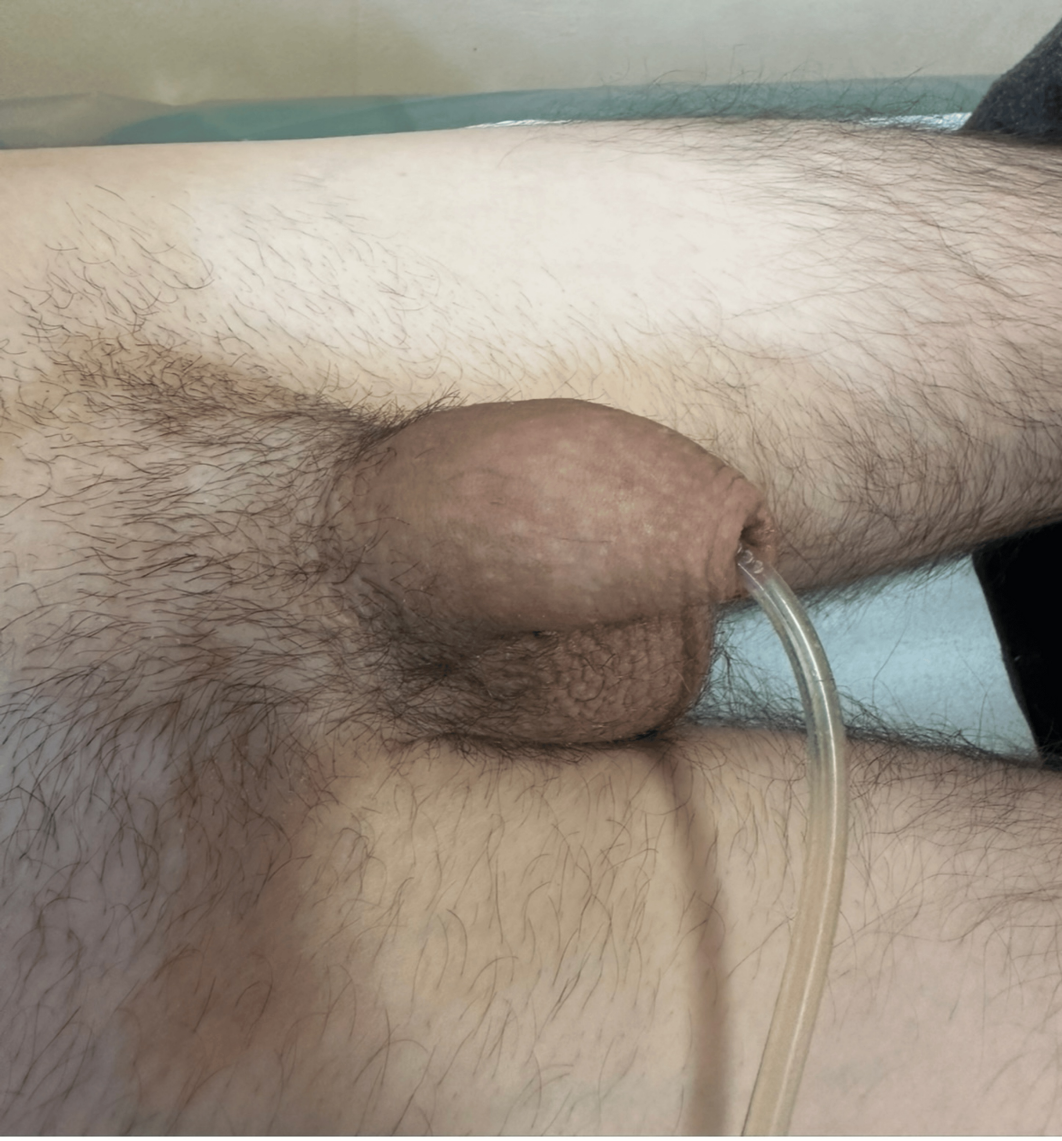
Postoperative day 14: appearance of external genitalia with Foley catheter in place. No signs of infection or hematoma

**Figure 3:**
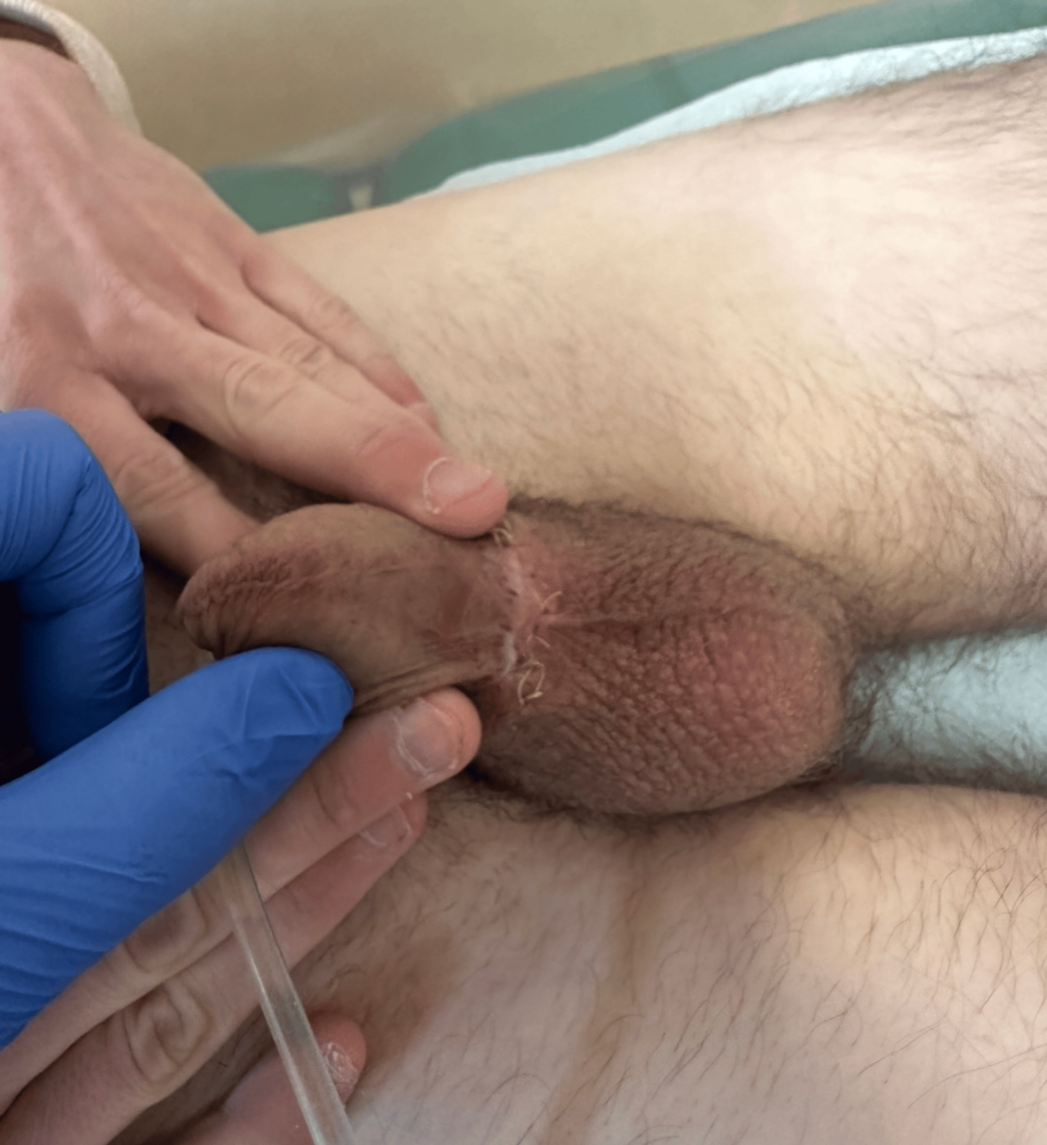
Postoperative day 14: satisfactory wound healing with normal anatomical appearance

The postoperative course was uneventful. The patient was discharged on postoperative day three with the catheter in place. At the 14-day follow-up, physical examination revealed satisfactory wound healing without signs of infection or hematoma (Figure 2 and Figure 3).

## Discussion

This case is notable due to the absence of classic signs of penile fracture, such as hematoma, palpable defect, or deformity. The isolated finding of urethrorrhagia may lead to underdiagnosis or delay in intervention. Surgeons should maintain a high index of suspicion even in the absence of typical external findings [1,2]. Penile fracture typically presents with a history of trauma during sexual activity, often accompanied by a snapping sound, pain, immediate detumescence, and swelling. While cavernosal rupture is the hallmark finding, associated urethral injury occurs in up to 20% of cases and should be suspected in the presence of urethrorrhagia or voiding difficulties [3,4]. In this case, early surgical intervention allowed for precise anatomical repair of both the urethra and cavernosal tissue. The penoscrotal approach provided optimal exposure. Intraoperative saline testing is a simple and effective method for identifying cavernosal defects. Early reconstruction reduces the risk of erectile dysfunction, curvature, and stricture formation [2,3]. This case highlights the importance of considering significant structural injury in patients with urethrorrhagia following intercourse and supports the role of early surgical exploration in suspected penile trauma.

## Conclusions

Combined urethral and cavernosal injury following intercourse is rare but serious. High clinical suspicion, timely diagnosis, and prompt surgical management are key to ensuring good outcomes. Penile exploration, anatomical repair, and layered closure can restore function and prevent complications.
